# Correlation Between Hemoglobin Levels with Muscle Function and the Risk of Sarcopenia in Patients with Cancer of the Digestive System: A Cross-Sectional Study

**DOI:** 10.3390/muscles5020042

**Published:** 2026-06-10

**Authors:** Elisa Silva Correia, Jéssika Martins Siqueira, Gustavo Duarte Pimentel

**Affiliations:** 1 Faculty of Medicine, Federal University of Goiás, Goiania 74605-050, GO, Brazil; elisasilva.nutri@gmail.com; 2 Faculty of Nutrition, Federal University of Goiás, Goiania 74605-080, GO, Brazil; jessikanutriufg@gmail.com

**Keywords:** cancer, anemia, hospitalized, sarcopenia, muscle strength

## Abstract

Hemoglobin levels play an important role in oxygen delivery to skeletal muscle, and reduced levels may impair muscle function and contribute to sarcopenia, particularly in patients with cancer. The aim of the present study was to evaluate the influence of hemoglobin levels on muscle mass and function in patients with digestive tract cancer. Methods: Patients of both sexes aged up to seventy years with cancers of the digestive system undergoing surgical and clinical treatment at an oncology referral center were included. Hemoglobin levels were assessed using a blood count, and the risk of sarcopenia was estimated using the Mini Sarcopenia Risk Assessment (MSRA). A total of 82 patients were evaluated, with a mean age of 55.4 years. Colon cancer was the most prevalent (41.6%), followed by rectal (18.3%) and stomach (14.6%) cancers. The risk of sarcopenia was estimated at 85.4%, and the prevalence of low hemoglobin levels was 71.9%; 35.4% of patients presented moderate hemoglobin depletion. Hemoglobin levels showed a moderate correlation with gait speed and a slight correlation with calf circumference, handgrip strength, and MSRA score. In conclusion, the risk of sarcopenia and low hemoglobin levels are present in patients with digestive tract cancer. Additionally, hemoglobin levels positively correlate with indicators of muscle function and the risk of sarcopenia.

## 1. Introduction

Cancer of the digestive system is highly relevant in the global epidemiological scenario due to its high incidence. This category includes tumors located in the gastrointestinal tract as well as in organs associated with digestion [1,2,3]. According to current estimates, neoplasms of the digestive system account for five of the ten most prevalent types of cancer worldwide, in both sexes [4].

In clinical practice, patients with digestive system cancers present greater alterations in nutritional and biochemical status compared to those with other types of tumors [5,6,7]. Therefore, comprehensive multidisciplinary follow-up is essential, both during clinical treatments such as chemotherapy and/or radiotherapy and in surgical management [5,7].

These patients are particularly susceptible to complications. In addition to metabolic alterations caused by cancer pathophysiology, several mechanisms contribute to this vulnerability. These include disruptions in energy metabolism and increased resting energy expenditure induced by both the tumor and the associated inflammatory state. Furthermore, patients often exhibit significant clinical manifestations related to tumor location [5]. Among these, mechanical obstruction of food passage, impaired enzyme secretion, and inadequate pH regulation stand out—mechanisms that are essential for proper food intake and nutrient metabolism [6,7,8].

As a consequence of these clinical manifestations and metabolic alterations, patients with digestive system cancers present a high prevalence of both sarcopenia and reduced hemoglobin (Hb) levels [9,10,11]. These conditions may occur concomitantly and are associated with worse prognosis, increased hospitalizations, reduced quality of life, and lower survival rates [12,13,14,15]. However, the relationship between low Hb levels and sarcopenia is not yet fully elucidated [16,17]. Evidence suggests that reduced Hb levels may lead to a hypoxic muscle microenvironment, negatively affecting protein synthesis and muscle regeneration [11,18,19].

This imbalance between anabolic and catabolic pathways is further exacerbated by circulating pro-inflammatory cytokines commonly elevated in cancer, such as interleukin-6 (IL-6), tumor necrosis factor-alpha (TNF-α), and C-reactive protein (CRP) [12,20,21]. Collectively, these factors may accelerate muscle mass loss, making it more difficult to preserve muscle strength and physical function in patients with digestive system cancers [12,21,22].

Given this context, it is essential to assess the risk and indicators of sarcopenia at all stages of treatment [5]. Among the available screening tools, the seven-item Mini Sarcopenia Risk Assessment (MSRA) stands out. This instrument includes variables that extend beyond strength and physical performance [23,24,25]. It also considers factors such as reduced food intake, consumption of protein-rich foods, and weight loss—elements well established in the literature as determinants of muscle mass [23,24,25]. Notably, the MSRA has already been adapted for use in hospitalized cancer populations [25].

For the assessment of sarcopenia indicators, calf circumference is used as a proxy for muscle mass, handgrip strength reflects muscle strength, and gait speed evaluates physical performance [26,27,28]. These tools are easily accessible in both clinical and research settings, and their use should be encouraged in the routine care of patients with digestive tract cancer [5,21].

Furthermore, considering the impact of low Hb levels on sarcopenia, incorporating blood count analysis as an additional screening tool for muscle impairment may represent a promising strategy for earlier and more effective interventions in patients with digestive system neoplasms [12,22]. It is important to note that complete blood count testing is low-cost and routinely performed in clinical and hospital settings that manage oncology patients [8,29].

Although anemia and sarcopenia frequently coexist in oncology patients, evidence specifically addressing the relationship between hemoglobin levels and sarcopenia indicators in patients with digestive system cancers remains limited. Therefore, the aim of this study was to evaluate the prevalence of low Hb levels and their correlation with the risk and indicators of sarcopenia in patients with cancer of the digestive system.

## 2. Methods

### 2.1. Study Design, Ethical Aspects, and Sampling

This was a cross-sectional study conducted at the Hospital das Clínicas of the Federal University of Goiás. The study was approved by the local Research Ethics Committee in 2021 under CAAE number 46717821.8.0000.5083. All participants received detailed information about the study procedures and provided written informed consent in accordance with the Declaration of Helsinki.

The study included male and female patients aged 18 years or older with primary cancer of the gastrointestinal tract or of organs and glands associated with the digestive system. Participants were receiving care in surgical and clinical units or undergoing outpatient chemotherapy at the aforementioned hospital. The following exclusion criteria were applied: exclusive palliative care, presence of amputations or anasarca, diagnosis of acquired immunodeficiency syndrome, stromal tumors, sepsis, and presence of mental confusion or cognitive impairment. The sample was probabilistic by convenience.

### 2.2. Variables of Interest

Clinical and socioeconomic data were collected from medical records and through interviews with participants. Information regarding diagnosis (tumor location and type) was obtained from medical records, and the International Classification of Diseases (ICD-10) was used to classify cancer types. During the interviews, data were collected on education level, self-reported race, current alcohol consumption and smoking status, use of nutritional supplements, and physical activity practices.

Nutritional status was assessed by measuring body weight and height to calculate body mass index (BMI), using specific cut-off points for adults and older individuals [30]. Muscle mass was estimated using calf circumference, applying appropriate cut-off points stratified by sex for adults and specific criteria for older individuals. Calf circumference is a simple, low-cost, and widely used anthropometric measure for screening reduced muscle mass in clinical settings. Although imaging-based methods such as computed tomography (CT), dual-energy X-ray absorptiometry (DXA), and bioelectrical impedance analysis (BIA) are considered reference standards for muscle mass assessment, they are not routinely available in all healthcare settings and often require specialized equipment and trained personnel. Therefore, calf circumference was used as a feasible proxy indicator of muscle mass in this clinical context. For classification, adjustments for BMI were applied, with reductions of 3 cm for BMI between 25 and 30 kg/m^2^ and 7 cm for BMI between 30 and 40 kg/m^2^ [26].

Data collection, as well as the assessment of nutritional status and muscle strength and function, was performed at a single time point by trained evaluators using standardized procedures. Hemoglobin levels were obtained from a complete blood count performed within the same week as the muscle function assessment.

### 2.3. Assessment of Muscle Function and Risk of Sarcopenia

Muscle function was assessed using dominant-hand handgrip strength and the four-meter gait speed test. Handgrip strength was measured using a Takei Physical Fitness Test^®^ dynamometer (TKK 5401 Grip-D, Smedley, Takei, Tokyo, Japan). For both assessments, the cutoff points proposed by the European Working Group on Sarcopenia in Older People were adopted [31].

To assess the risk of sarcopenia, the seven-item Mini Sarcopenia Risk Assessment (MSRA-7) was used [23,24]. This is an easy-to-administer tool that has been translated and adapted for hospitalized cancer patients [25]. It consists of questions that generate individual scores, which are summed to obtain a final classification score. The instrument includes variables such as muscle function, weight loss, number of meals per day, and consumption of protein-rich food groups. A risk of sarcopenia was defined as a score ≤ 30 points.

### 2.4. Hemoglobin Assessment

Hemoglobin levels were assessed using hemoglobin concentration (Hb), expressed in grams per deciliter (g/dL), obtained from complete blood count results recorded in the patients’ medical records. The classification of hemoglobin levels as normal or reduced was based on the diagnostic criteria for anemia established by the World Health Organization, considering reduced Hb levels as <13.0 g/dL for men and <12.0 g/dL for women [32].

The stratification of anemia severity also followed World Health Organization criteria, with Hb values < 8.0 g/dL classified as severe anemia, values between 8.0 and 10.9 g/dL as moderate anemia, and values ≥ 11.0 g/dL as mild anemia for both sexes [32].

### 2.5. Statistical Analysis

Statistical analyses were performed using RStudio version 4.4.1. The normality of continuous variables was assessed using the Shapiro–Wilk test. Results are presented as absolute values (mean and standard deviation or median and interquartile range) and relative frequencies (percentages).

Participants were categorized into two groups (normal Hb and low Hb) for comparisons of all study variables. Subsequently, participants with low Hb levels were stratified according to severity, and the frequency of cancer type, risk of sarcopenia, and adequacy of calf circumference, handgrip strength, and gait speed were estimated for each severity category.

Spearman’s correlation analysis was performed to evaluate the associations between hemoglobin levels and muscle mass, muscle function, and risk of sarcopenia. For all analyses, the level of statistical significance was set at *p* < 0.05.

## 3. Results

A total of 82 patients with digestive system cancer admitted to clinical or surgical units, as well as the outpatient chemotherapy clinic, were evaluated during 2023 and the first half of 2024. The mean age was 55.4 ± 9.6 years, and the majority were men (63.41%) and of mixed race (56.09%).

Regarding lifestyle habits, patients with low Hb levels showed a higher prevalence of smoking and alcohol consumption and were more physically active; however, no statistically significant differences were observed between the groups (Table 1).

The most prevalent types of cancer in the sample were colon cancer (41.46%), followed by rectal and stomach cancers (18.29% and 14.63%), respectively. The prevalence of low Hb levels was 71.95%, of which 32.2% were classified as mild, 49.15% as moderate, and 18.64% as severe. A statistically significant difference was observed between groups when comparing the presence or absence of low Hb levels according to tumor location (*p* = 0.01).

Regarding oral nutritional therapy and meal frequency, a high prevalence of supplementation (64.6%) and consumption of three or more meals per day (65.85%) was observed. Clinical variables are presented in Table 2.

Assessment of nutritional status revealed a mean BMI within the normal range (23.14 ± 5.04 kg/m^2^); however, approximately one-third of the patients were underweight. Additionally, indicators of muscle mass and function were below recommended values for the respective sex and age groups. Statistically significant differences were observed in mean calf circumference, handgrip strength, and gait speed when comparing patients with and without low Hb levels.

The risk of sarcopenia was present in 85.37% of the patients. Among patients with low Hb levels, 74.29% were classified as being at risk of sarcopenia, compared with 25.71% among those with normal Hb levels (Table 2).

Patients with low Hb levels also presented greater alterations in hematological indices from the complete blood count and higher neutrophil-to-lymphocyte ratio (NLR), with statistically significant differences between groups (Table 3).

**Figure 1:**
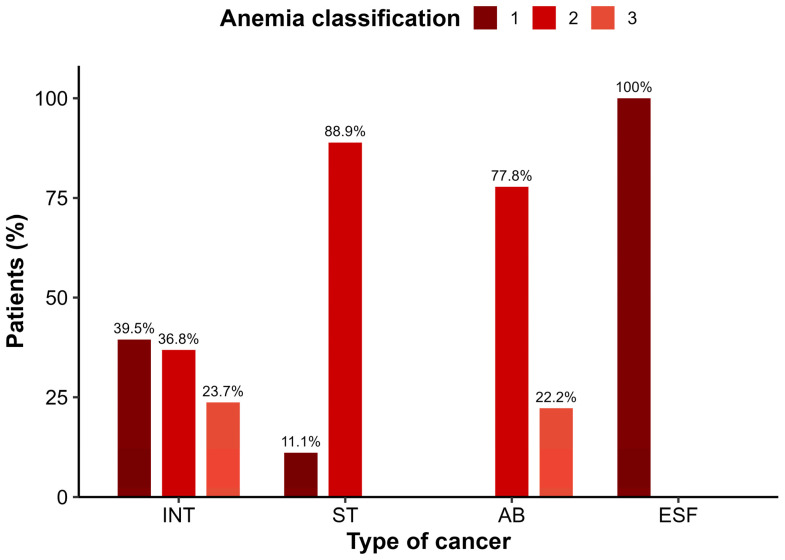
Distribution of hemoglobin depletion severity by cancer type. Legend: 1: Mild depletion; 2: Moderate depletion; 3: Severe depletion. INT: cancers of the intestines and rectum; ST: stomach cancer; AB: cancers of organ and adnexal glands; ESF: esophageal cancer.

When cancer types were stratified according to anemia severity, a higher prevalence of moderate Hb depletion was observed in stomach cancers and cancers of glands/adnexal organs, whereas cancers of the intestine (small intestine, colon, and rectum) and esophagus showed a higher prevalence of mild depletion (*p* = 0.004) (Figure 1).

**Figure 2:**
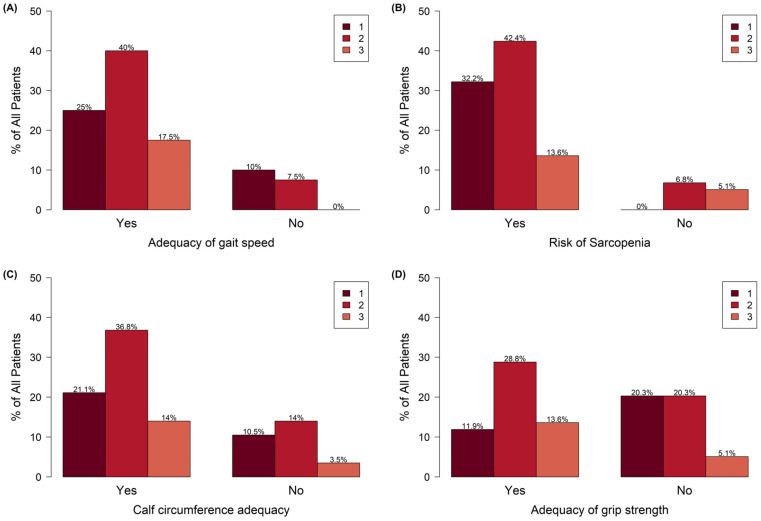
Distribution of hemoglobin depletion severity by indicators of muscle mass, muscle function, and risk of sarcopenia. Legends: (**A**) Gait speed; (**B**) Sarcopenia risk assessed by the Mini Sarcopenia Risk Assessment (MSRA7); (**C**) Calf circumference; (**D**) Handgrip strength. 1: Mild depletion; 2: Moderate depletion; 3: Severe depletion. Yes: Inadequate; No: Suitable.

Figure 2 illustrates the distribution of Hb depletion severity in relation to gait speed, risk of sarcopenia, calf circumference, and handgrip strength. However, no statistically significant differences were observed between the groups for these outcomes (gait speed, *p* = 0.257; risk of sarcopenia, *p* = 0.075; calf circumference, *p* = 0.750; handgrip strength, *p* = 0.131).

**Figure 3:**
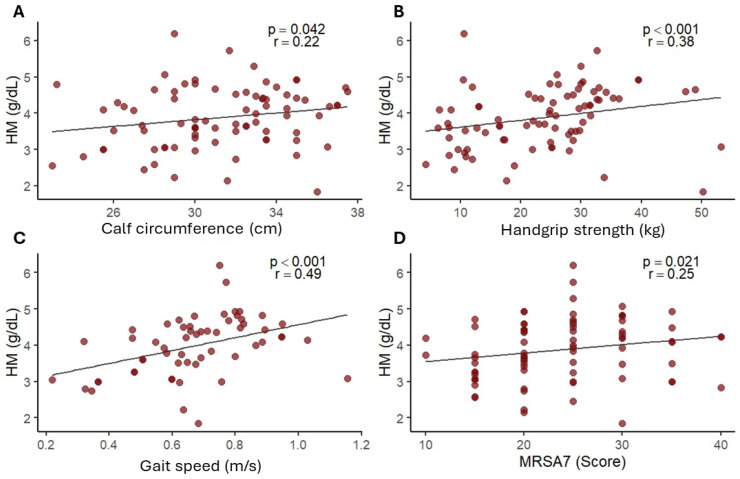
Spearman’s correlation between hemoglobin levels and indicators of muscle mass, muscle function, and risk of sarcopenia. Legenda: r: *rho de Spearman*; HM: hemoglobin; (**A**) Calf circumference; (**B**) Handgrip strength; (**C**) Gait speed; (**D**) MRSA: Mini Sarcopenia Risk Assessment.

Spearman’s correlation analysis between Hb levels and variables related to muscle mass and function showed weak but significant positive correlations with calf circumference (r = 0.22; *p* = 0.042), handgrip strength (r = 0.38; *p* < 0.001), and MSRA-7 score (r = 0.25; *p* = 0.021). A moderate positive correlation was also observed with gait speed (r = 0.49; *p* < 0.001) (Figure 3).

## 4. Discussion

In the present study, the sample consisted predominantly of adults and men. Regarding tumor location, intestinal and stomach cancers were the most prevalent. Additionally, the high prevalence of sarcopenia and low Hb levels observed is concerning.

There is a growing incidence of digestive system cancers among adults compared to older populations. This trend may be attributed to the adoption of unhealthy behaviors, such as physical inactivity, alcohol consumption, and smoking, which are well-established risk factors in the etiopathogenesis of these cancers [2,3,20]. The sample characteristics observed in this study, with a higher prevalence of intestinal and stomach tumors, are consistent with national epidemiological data [33].

However, it is important to highlight that disease occurrence in the adult population may negatively impact socioeconomic development, as adults represent the primary economically productive group [1,3,4]. Furthermore, despite the increasing incidence of digestive system cancers among younger individuals, societies are undergoing a demographic transition toward population aging. Consequently, cancer survivors may impose an additional burden on healthcare systems due to the long-term management of disease-related sequelae [1,34].

Although all evaluated patients were either hospitalized or undergoing outpatient chemotherapy, smoking and alcohol consumption were still reported in approximately 20.0% of the sample. Among individuals who smoked or consumed alcohol, the majority presented low hemoglobin (Hb) levels. Alcohol and tobacco use can generate significant amounts of reactive oxygen species and increase circulating pro-inflammatory cytokines, such as nuclear factor kappa B (NF-κB), interleukin-6 (IL-6), and tumor necrosis factor-alpha (TNF-α). In addition to affecting the tumor microenvironment, these factors may exacerbate systemic inflammation [4,35]. Systemic inflammation and oxidative stress are directly associated with the development of anemia and sarcopenia in patients with cancer [11,12,18,29].

Inflammation is a key component of cancer pathophysiology. Tumor-induced alterations in the microenvironment lead to systemic inflammation, which contributes to the catabolism of various tissues, primarily through proteolysis and gluconeogenesis. This process also promotes anorexia and alters immune function and erythropoiesis [20,36,37,38].

To assess the inflammatory status of cancer patients, the neutrophil-to-lymphocyte ratio (NLR) has been widely proposed. In the present study, patients exhibited elevated NLR values. When comparing groups, patients with hemoglobin (Hb) depletion showed NLR values nearly twice as high as those with normal Hb levels. The literature indicates that an NLR greater than three is associated with worse outcomes, including reduced survival and a higher risk of metastasis, in patients with digestive cancers and other malignancies [8,39,40]. Therefore, elevated NLR may represent one of the factors contributing to the high prevalence of sarcopenia risk and low Hb levels observed in this study.

The nutritional status of the sample, assessed by mean BMI, indicated an overall classification within the normal range. However, approximately one-third of the patients were underweight. In addition, indicators of muscle mass (calf circumference) and muscle function (handgrip strength and gait speed) were below recommended values. These indicators were also significantly lower in patients with low Hb levels. These findings support previous literature emphasizing the importance of incorporating muscle health assessments into the routine care of patients with gastrointestinal cancers. Notably, reduced muscle strength and impaired physical performance may occur even in individuals with normal BMI [8,38]. Failure to identify muscle impairment may delay timely and appropriate interventions.

This may result in reduced quality of life, worse prognosis due to complications, and increased chemotherapy-related toxicity [10,41,42,43]. Handgrip dynamometry is considered the most validated method for assessing muscle strength. It is recommended by clinical guidelines and widely accepted by healthcare professionals, reinforcing the importance of incorporating this assessment into routine care for patients with gastrointestinal cancer [8,10,22,44].

Low Hb levels were observed in 71.9% of the patients, and approximately half of these individuals presented moderate depletion. According to the MSRA-7 questionnaire, 85.4% of the patients were at risk of sarcopenia. Among these individuals, 74.3% had low Hb levels. A high prevalence of sarcopenia risk and low Hb levels has been reported in previous studies involving patients with digestive system cancers, although these conditions are often evaluated separately. Reported prevalence ranges from 63.0% to 77.5% for low Hb levels and from 78.0% to 91.0% for sarcopenia risk [13,18,24,25,45,46]. These findings reinforce the substantial burden of these comorbidities among patients with digestive system cancers.

When evaluating the relationship between Hb levels and sarcopenia indicators, our study found significant positive correlations between Hb levels and calf circumference, handgrip strength, gait speed, and MSRA-7 scores. Cho Lee and Kang (2024) [12] also identified a positive correlation between the skeletal muscle index derived from CT scans and Hb levels in patients with colorectal cancer. Studies including patients with various cancer types have similarly reported associations between low Hb levels, reduced handgrip strength, and poorer performance status [9,13].

Population-based cohort studies conducted in older adults with a mean age of approximately 70 years have also investigated the influence of Hb depletion on sarcopenia and its indicators, proposing Hb cut-off values for these outcomes. However, the prevalence of low Hb levels in these populations ranged from approximately 14.0% to 16.0% [16,46], which is considerably lower than that observed in studies involving cancer patients [1,12,25,44]. This discrepancy highlights the limitations of generalizing these cut-off values to oncology populations and underscores the need for studies establishing specific thresholds for patients with gastrointestinal cancers.

The main pathophysiological mechanisms linking low Hb levels and sarcopenia involve systemic inflammation [8,11,37,40]. Pro-inflammatory cytokines promote protein breakdown and muscle loss, contributing to the development of sarcopenia. Inflammatory markers such as C-reactive protein, interleukin-6 (IL-6), and tumor necrosis factor (TNF) are often elevated in anemic patients. At the same time, the inflammatory state impairs erythropoiesis and disrupts iron metabolism, contributing to anemia. Consequently, these mechanisms may create a feedback loop that perpetuates both conditions [8,12,16,17,20].

Hb plays a fundamental role in oxygen delivery to tissues. Its depletion can lead to musculoskeletal hypoxia, resulting in fatigue, reduced muscle strength, and impaired muscle regeneration. These changes may predispose individuals to more sedentary behavior, which further contributes to the development of sarcopenia [18,29,44]. This mechanism represents one of the main hypotheses linking Hb levels to muscle health and physical performance in patients with cancer. Considering both the literature and the findings of the present study, our results highlight the potential utility of routine complete blood count testing as a complementary tool in the evaluation of muscle health in patients with digestive system cancers.

The present study has some limitations. These include the relatively small sample size and the inclusion of patients from a single cancer referral center. In addition, biochemical assessments of iron status and detailed dietary intake data were not available. These factors may influence the pathophysiology of hematological alterations and nutritional status, which are closely related to the risk of sarcopenia.

Furthermore, the assessment of muscle mass using only calf circumference, without a reference method, represents an additional limitation. Although techniques such as dual-energy X-ray absorptiometry (DXA) and computed tomography are considered gold-standard methods, they are not readily available in all healthcare settings and require specialized training for accurate interpretation. To minimize potential bias associated with the use of calf circumference, standardized measurement procedures were adopted, and sex- and age-specific cutoff points were applied. Additionally, measurements were adjusted for BMI to improve accuracy.

Another limitation is the cross-sectional design and the relatively small sample from a single center, which may limit the generalizability of the findings. Moreover, potentially relevant variables—such as cancer stage, treatment modality, inflammatory markers, nutritional status, and iron levels—were not available for analysis and may have influenced the observed associations. Therefore, future studies with larger samples and more comprehensive clinical data are needed to further investigate these relationships.

As strengths, this study addresses a gap in the literature regarding the relationship between Hb depletion and the risk of sarcopenia in patients with digestive system cancers. Additionally, the assessment tools used are simple, accessible, and easily applicable in clinical practice, facilitating replication of the study and supporting the implementation of these findings in healthcare settings that manage patients with gastrointestinal cancers.

## 5. Conclusions

Hemoglobin (Hb) levels were positively associated with the risk of sarcopenia and with indicators of muscle mass, muscle strength, and physical performance in patients with digestive system cancer. Therefore, incorporating complete blood count assessment into the evaluation of sarcopenia-related indicators may facilitate earlier diagnosis and enable more effective interventions, ultimately improving prognosis and quality of life in this population.

## Figures and Tables

**Table 1:** Sociodemographic and lifestyle characterization of patients with digestive tract cancer.

		Low Hemoglobin Level		
Variable	Total (n: 82)	Yes (n: 59)	No (n: 23)	*p*
Age	55.4 (±9.6) ^a^	54.9 (±8.7) ^a^	56.8 (±11.2) ^a^	0.220 ^b^
Sex	% (n)	% (n)	% (n)	
Male	63.4 (52)	71.2 (37)	28.8 (15)	1 ^c^
Female	36.6 (30)	73.3 (22)	26.7 (8)	
Education				
Illiterate	10 (8)	50 (4)	50 (4)	0.694 ^d^
Incomplete elementary school	43.7 (35)	74.3 (26)	25.7 (9)	
Elementary school	18.7 (15)	73.4 (11)	26.6 (4)	
High school	20 (16)	68.7 (11)	31.3 (5)	
Higher education	7.5 (6)	83.3 (5)	16.4 (1)	
Race				
White	34.1 (28)	75 (21)	25 (7)	0.76 ^d^
Brown	56.1 (46)	71.7 (33)	28.3 (13)	
Black	9.8 (8)	62.5 (5)	37.5 (3)	
Smoking				
No	81.7 (67)	71.6 (48)	28.4 (19)	1 ^d^
Yes	18.3 (15)	73.3 (11)	26.7 (4)	
Alcoholism				
No	76.8 (63)	74.6 (47)	25.4 (16)	0.49 ^c^
Yes	23.2 (19)	63.2 (12)	36.8 (7)	
Physical activity				
No	78.1 (64)	71.9 (46)	28.3 (18)	1 ^c^
Yes	21.9 (18)	72.2 (13)	27.8 (5)	

Legends: ^a^ Result expressed as mean and standard deviation; ^b^ Wilcoxon test; ^c^ Chi-square test; ^d^ Fisher’s exact test.

**Table 2:** Clinical characterization, nutritional status, muscle function and sarcopenia risk classification in patients with digestive tract cancer.

		Low Hemoglobin Level		
Variable	Total (n: 82) % (n)	Yes (n: 59) % (n)	No (n: 23) % (n)	*p*
Localization of cancer				
Esophagus	3.6 (3)	100 (3)	0	0.01 ^a^
Stomach	14.6 (12)	75 (9)	25 (3)	
Small intestine	3.6 (3)	66.7 (2)	33.3 (1)	
Colon	41.6 (34)	85.3 (29)	14.7 (5)	
Straight	18.3 (15)	46.7 (7)	53.3 (8)	
Gallbladder	4.9 (4)	25 (1)	75 (3)	
Liver	7.3 (6)	50 (3)	50 (3)	
Pancreas	6.1 (5)	100 (5)	0	
Admission				
Surgical clinic	58.5 (48)	72.9 (35)	27.1 (13)	0.58 ^a^
Medical clinic	18.3 (15)	80 (12)	20 (3)	
Chemotherapy ambulatory	23.2 (19)	63.2 (12)	36.8 (7)	
Use of nutritional supplement				
No	64.6 (53)	67.9 (36)	3.1 (17)	0.40 ^b^
Yes	35.4 (29)	79.3 (23)	20.7 (6)	
Number of meals per day				
<3 meals	34.2 (28)	67.9 (19)	32.2 (9)	0.74 ^b^
≥3 meals	65.8 (54)	74.1 (40)	25.9 (14)	
Low Hb level	71.9 (59)	100 (59)	-	
Mild	23.2 (19)	32.2 (19)	-	
Moderate	35.4 (29)	49.2 (29)	-	
Severe	13.4 (11)	18.6 (11)	-	
Weight	64.3 (±15.3) ^a^	63.2 (±16.4) ^a^	67.4 (±12) ^a^	0.138 ^b^
BMI (kg/m^2^)	23.1 (±5) ^a^	23.2 (±5.6) ^a^	23.1 (±3.5) ^a^	0.804 ^b^
Low weight	28 (23)	73.9 (17)	26.1 (6)	0.742 ^c^
Eutrophic	47.6 (39)	66.7 (26)	33.3 (13)	
Overweight	20.7 (17)	76.48 (13)	23.5 (4)	
Obesity	3.7 (3)	100 (3)	0	
Calf circumference (cm) *	31.2 (±3.5) ^a^	30.6 (±3.6) ^a^	32.9 (±2.4) ^a^	0.001 ^d^
Low	63.8 (51)	80.4 (41)	19.6 (10)	0.032 ^e^
Adequate	36.2 (29)	55.2 (16)	44.8 (13)	
Handgrip strength (kg) *	23.6 (±10.9) ^a^	21.9 (±10.8) ^a^	27.9 (±10.3) ^a^	0.007 ^b^
Low	45.1 (37)	86.5 (32)	13.5 (5)	0.01 ^e^
Adequate	54.9 (45)	60 (27)	40 (18)	
Gait speed (m/s) **	0.67 (±0.2) ^a^	0.62 (±0.2) ^a^	0.77 (±0.1) ^a^	<0.001 ^d^
≤0.8 m/s	72.4 (42)	78.6 (33)	21.4 (9)	0.024 ^e^
>0.8 m/s	27.6 (16)	43.7 (7)	56.3 (9)	
MRSA7	23.6 (±7) ^a^	22.8 (±6.8) ^a^	25.7 (±7.4) ^a^	0.087 ^b^
Risk of Sarcopenia	85.4 (70)	74.3 (52)	25.7 (18)	0.430 ^e^
No risk	14.6 (12)	58.3 (7)	41.67 (5)	

Legends: ^a^ Result expressed as mean and standard deviation. ^b^ Wilcoxon test, ^c^ Fisher’s exact test, ^d^ *t*-test, ^e^ Chi-square test. * n: 81 observations. ** n: 58 observations; BMI: body mass index; MRSA7: Mini Sarcopenia Risk Assessment.

**Table 3:** Blood count values of the total sample and stratified by the presence of low hemoglobin.

		Low Hemoglobin Level		
Variable	Total (n: 82)	Yes (n: 59)	No (n: 23)	*p*
Erythrocytes (t/L)	3.86 ± 0.83	3.62 ± 0.80	4.48 ± 0.51	<0.001 ^a^
Hemoglobin (g/dL)	11.26 ± 3.85	9.92 ± 1.80	14.71 ± 5.37	<0.001 ^b^
Hematocrit (%)	32.81 ± 7.07	30.29 ± 5.44	39.30 ± 6.72	<0.001 ^b^
Mean Corpuscular Volume (fL)	88 (82.82–92.9) **	86.7 (79.4–92.8) **	89.5 (85.7–94.7) **	0.119 ^b^
Mean Corpuscular Hemoglobin (pg)	29.06 ± 3.24	28.38 ± 3.16	30.81 ± 2.82	0.001 ^a^
Mean Corpuscular Hemoglobin Concentration (g/dL)	32.90 ± 1.24	32.67 ± 1.22	33.47 ± 1.11	0.006 ^a^
Red Cell Distribution Width (%)	15.84 ± 4.17	16.59 ± 4.57	13.92 ± 1.86	<0.001 ^b^
Platelets (/μL)	249.561 ± 135.414	26.094 ± 149.475	212.913 ± 82.649	0.185 ^b^
Leukocytes(/μL)	7.030 (4.610–9.767) **	7.720 (5.190–10.490) **	5.840 (3.920–8.045) **	0.110 ^b^
Lymphocytes (/μL)	1.431 ± 988	1.423 ± 1.136	1.449 ± 450	0.171 ^b^
Neutrophils (/μL)	6.118 ± 5.712	6.670 ± 6.360	4.725 ± 3.316	0.201 ^b^
NLR	5.68 ± 4.43	6.38 ± 5.84	3.89 ± 2.60	0.011 ^b^

Legends: ^a^ *t*-test, ^b^ Wilcoxon test. ** Result expressed as median and interquartile. NLR: Neutrophils lymphocytes ratio.

## Data Availability

The original contributions presented in this study are included in the article. Further inquiries can be directed to the corresponding author.
